# In-vivo assessment of the morphology and hemodynamic functions of the BioValsalva™ composite valve-conduit graft using cardiac magnetic resonance imaging and computational modelling technology

**DOI:** 10.1186/s13019-014-0193-6

**Published:** 2014-12-09

**Authors:** Emaddin Kidher, Zhuo Cheng, Omar A Jarral, Declan P O’Regan, Xiao Yun Xu, Thanos Athanasiou

**Affiliations:** The Department of Surgery and Cancer, Imperial College London, St Mary’s Hospital, London, W2 1NY UK; Department of Chemical Engineering, Imperial College London, South Kensington, London, SW7 2AZ UK; Institute of Clinical Science, Imperial College London, Hammersmith Hospital, London, W12 0HS UK

**Keywords:** Composite valve-conduit, Aortic valve, Aortic root, Aortic prosthesis, BioValsalva, Computational fluid dynamic

## Abstract

**Background:**

The evaluation of any new cardiac valvular prosthesis should go beyond the classical morbidity and mortality rates and involve hemodynamic assessment. As a proof of concept, the objective of this study was to characterise for the first time the hemodynamics and the blood flow profiles at the aortic root in patients implanted with BioValsalva™ composite valve-conduit using comprehensive MRI and computer technologies.

**Methods:**

Four male patients implanted with BioValsalva™ and 2 age-matched normal controls (NC) underwent cardiac magnetic resonance imaging (MRI). Phase-contrast imaging with velocity-mapping in 3 orthogonal directions was performed at the level of the aortic root and descending thoracic aorta. Computational fluid dynamic (CFD) simulations were performed for all the subjects with patient-specific flow information derived from phase-contrast MR data.

**Results:**

The maximum and mean flow rates throughout the cardiac cycle at the aortic root level were very comparable between NC and BioValsalva™ patients (541 ± 199 vs. 567 ± 75 ml/s) and (95 ± 46 vs. 96 ± 10 ml/s), respectively. The maximum velocity (cm/s) was higher in patients (314 ± 49 vs. 223 ± 20; *P* = 0.06) due to relatively smaller effective orifice area (EOA), 2.99 ± 0.47 vs. 4.40 ± 0.24 cm^2^ (*P* = 0.06), however, the BioValsalva™ EOA was comparable to other reported prosthesis. The cross-sectional area and maximum diameter at the root were comparable between the two groups. BioValsalva™ conduit was stiffer than the native aortic wall, compliance (mm^2^ • mmHg^−1^ • 10^−3^) values were (12.6 ± 4.2 vs 25.3 ± 0.4.; *P* = 0.06). The maximum time-averaged wall shear stress (Pa), at the ascending aorta was equivalent between the two groups, 17.17 ± 2.7 (NC) vs. 17.33 ± 4.7 (BioValsalva™ ). Flow streamlines at the root and ascending aorta were also similar between the two groups apart from a degree of helical flow that occurs at the outer curvature at the angle developed near the suture line.

**Conclusions:**

BioValsalva™ composite valve-conduit prosthesis is potentially comparable to native aortic root in structural design and in many hemodynamic parameters, although it is stiffer. Surgeons should pay more attention to the surgical technique to maximise the reestablishment of normal smooth aortic curvature geometry to prevent unfavourable flow characteristics.

**Electronic supplementary material:**

The online version of this article (doi:10.1186/s13019-014-0193-6) contains supplementary material, which is available to authorized users.

## Background

Aortic root aneurysm, dissection and endocarditis associated with aortic valve dysfunction may be the most common examples of pathological conditions that often need concomitant replacement of aortic valve, aortic root and ascending aorta [1]. Traditionally, the modified Bentall technique with mechanical valve conduit is the standard surgical procedure for such pathologies with excellent short and long-term results [2],[3]. The main drawback of the classical modified Bentall procedure is the use of mechanical valve which mandate the use of anticoagulants. In addition, the increasing number of elderly patients requiring modified Bentall procedure make the use of biological composite valve-conduit more appealing option. Hemodynamics is an essential aspect in designing any vascular or valve product. Stentless aortic valve prosthesis seems to have better hemodynamic performance than stented valves [4] and may improve coronary circulation up to the expected normal level [5]. Stentless homografts have very good hemodynamic performance and durability but their use is limited by their availability [6], therefore stentless xenografts were designed and introduced to overcome these problems and maintain good hemodynamic profile similar to homograft aortic valves, with excellent mid-term results [7]. Although stentless xenografts roots offer good choice and satisfactory outcome, their use is limited by their length as they cannot be employed when the ascending aorta needs to be replaced unless Dacron graft extension is sutured [1]. This makes the procedure more complex, increasing the ischemic time and risk of bleeding from suture line.

Another important hemodynamic aspect in aortic valve prosthesis design is the presence or absence of sinuses of Valsalva [8]. Sinuses of Valsalva reduce the stress in the leaflet significantly, facilitate vortex formation that seems to help in smooth valve closure with less bending deformation in the longitudinal direction, and reduce the stress on the coronary anastomosis [8]-[10]. All the above factors can affect the durability and performance of the aortic bio-prosthetic valves, however, the effect of sinuses of Valsalva on coronary circulation is still debatable [11],[12].

All the above-mentioned limitations accelerated the designing and introduction of a new prefabricated composite bio-prosthetic valve–conduit graft (BioValsalva™) into the valve- conduit market since 2006. The BioValsalva™ (Vascutek Terumo, Renfrewshire, Scotland) is a composite valve-conduit graft which consists of stentless porcine aortic valve (elan valve™) pre-sewn to self-sealing trilaminated vascular graft (Vascutek Triplex™). It is a one-piece constructed graft making the procedure technically less complex, reducing cross-clamp and by-pass time and providing better haemostasis.

Preliminary data from Europe have been published [13]-[16] demonstrating good early post-operative results with low mortality rate and superior haemostatic properties. However, until date, no study has been conducted to assess the hemodynamic performance of the new generation of BioValsalva™ using advanced MRI and computer technologies. Cardiovascular magnetic resonance imaging (CMR) with phase-contrast velocity mapping and subsequent offline computational fluid dynamics (CFD) processing is a state-of-art effective imaging tool for the visualization of the streamlines and particle traces of the blood flow within the aortic root and thoracic aorta [17],[18].

The objective of the study was to assess the hemodynamics and to characterise the blood flow profiles in patients with implanted BioValsalva™ composite valve-conduit, and compare these with matched normal controls based on detailed analysis of cardiac MRI data and patient-specific computational flow modelling as a proof of concept assessment.

## Methods

Ethical approval (10/H0717/45, North London Research Ethic Committee 1) was granted and informed consent was obtained from all participants prior to inclusion. All patients with implanted BioValsalva™ pre-assembled composite valve-conduit graft were eligible. Age and gender matched healthy controls were recruited as normal control group (NC).

### Cardiovascular magnetic resonance imaging

MRI for patients was performed after the recommended minimum safety period of four weeks post-operatively, mean (49 ± 11 days). A Philips Achieva 1.5 T scanner (Philips Achieva, Royal Philips, Amsterdam, The Netherlands) with a 32 channel cardiac coil was used for all studies. Two-dimensional phase contrast imaging was performed in the aortic root, at the level of the annulus, and in the descending thoracic aorta at the level of the pulmonary bifurcation. Double oblique sections were obtained to image the aorta in cross-section. The velocity-encoding value was chosen as 10% above peak velocity in the direction measured and 100 phases per cardiac cycle were obtained. Reconstructed voxel size was 1.4 × 1.4 × 10 mm. Anatomic imaging of the thoracic aorta was achieved using a navigator-gated balanced steady state free precession angiogram with a reconstructed voxel size of 0.5 × 0.5 × 2 mm.

### Image analysis

#### Morphological parameters

**Figure 1:**
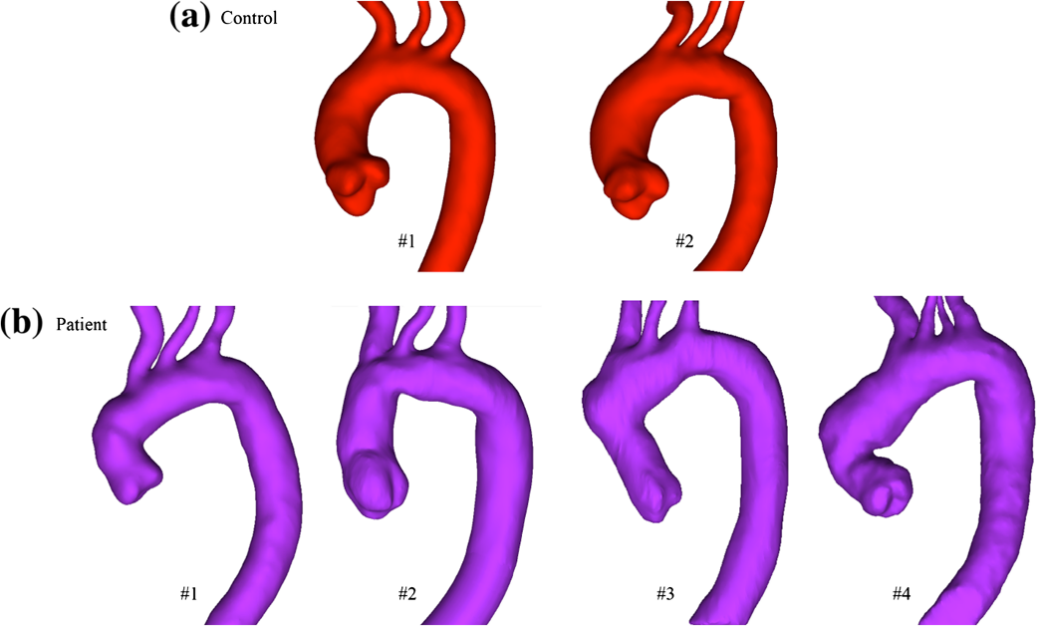
**Three-dimensional geometries of the aorta for all subjects.** **(a)** Normal control group and **(b)** BioValsalva™ patients group. The normal smooth curvature of the aorta is lost in BioValsalva^TM^ patients.

The three-dimensional geometry of aorta was reconstructed from the anatomic MR images in an image processing package Mimics (Materialise HQ, Louvain, Belgium). Each geometry model is starting from the aortic root to the middle of descending aorta at the level of diaphragm with the three branches on the arch included (Figure 1). The morphological parameters were calculated based on the reconstructed geometry of the aorta. The mean cross-sectional area and maximum diameter of the ascending aorta were measured along the fitted centreline of the aorta from the aortic root to the arising point of the brachiocephalic artery. The anatomic cross-sectional area of the aortic sinus (*A*_*sinus*_) was obtained at the middle of the aortic root, and the diameter of the sinus (*D*_*sinus*_) was determined from the cross-sectional area as

1 $$D_{\mathrm{sinus}}=2\sqrt{A_{\mathrm{sinus}}/\pi }$$

#### Hemodynamic parameters

Two -dimensional cine phase-contrast MRI of blood flow was performed in three orthogonal directions: feet-head, left-right and anterior-posterior separately on the selected imaging planes. The phase contrast images consisted of magnitude and phase images in pairs, and the magnitude images were utilized to identify and segment the anatomic region of the aortic root. The segmented region was then mapped to the corresponding phase image to calculate the velocity values in reference to encoded flow velocity. Velocity mapping was performed for all three components, and was converted to the through-plane as well as in-plane velocity maps over the cross section of aortic root. By integrating the through-plane velocity map over the cross-sectional area, the volumetric flow was calculated at each of the 100 time points; then the temporal volumetric flow waveform was reconstructed for a complete cardiac cycle. In this study, the temporal maximum volumetric flow rate (*Q*_*max*_), the time-averaged volumetric flow rate (*Q*_*mean*_) and the spatial maximum velocity at peak flow rate (*V*_*max*_) were examined. Additionally, the effective orifice area, EOA (*A*_*orifice*_) was calculated, which is defined as the area where the velocities are larger than the spatial averaged velocity at peak flow rate [18]. The ratio of the EOA to sinus area (*A*_*orifice*_*\A*_*sinus*_) was also calculated to further assess the function of aortic valves in both control and patient groups. A MATLAB program (The MathWorks, Inc, Natick, MA, USA) was developed to carry out all the analysis of phase contrast MR images in this study.

#### Aortic wall compliance

The blood pressure at the root of aorta was measured by a novel device Pulsecor (Pulsecor Ltd, Auckland, New Zealand) that estimates central aortic pressures from the brachial cuff pressure fluctuations [19]. It employs a physics-based model involving pressure wave reflection simulation to reconstruct the central pressure waveform according to the small oscillations of the intra-arterial pressure [19]. The Pulsecor device has been evaluated to be an accurate non-invasive method to estimate central aortic pressure [20]. The pressure of each subject was measured by Pulsecor device just before their MR scans, and the derived maximum systolic (*P*_s_) and minimum diastolic (*P*_d_) pressure were utilized for the calculation of compliance, distensibility and wall stiffness of the aortic root.

Segmentation of the magnitude images at the aortic root provides cross-sectional area changes throughout a cardiac cycle. The maximum systolic (*A*_*s*_) and minimum diastolic (*A*_*d*_) aortic root area were selected, and the systolic (*D*_*s*_) and diastolic (*D*_*d*_) diameter of the aortic root were determined by the area with the same correlation as equation (1). On the basis of the area and diameter, the wall compliance, distensibility and stiffness index β of the aortic root can be calculated as follows [21]:

2 $$\mathrm{Compliance}=\frac{A_{s}-A_{d}}{P_{s}-P_{d}}$$

3 $$\mathrm{Distensibility}=\frac{\left(A_{s}-A_{d}\right)/A_{d}}{P_{s}-P_{d}}$$

4 $$\mathrm{Stiffness}\;\mathrm{Index}\;\beta =\frac{\ln\left(P_{s}/P_{d}\right)}{D_{s}-D_{d}}$$

#### Computational fluid dynamic analysis

Based on the reconstructed three-dimensional geometry of the aortas, computational fluid dynamic (CFD) models were built for all the subjects in normal and patient groups. The patient-specific flow information obtained from phase-contrast MR data and the aortic pressure data measured with the Pulsecor device was applied to the computational model as boundary conditions. The three-directional encoded velocity profiles were mapped to the cross section of the aortic root in the model both spatially and temporally. The aortic pressure waveform was applied to the model outlet at the middle of descending aorta. The flow exiting through the three branches on the aortic arch was determined by the flow difference between the two MR imaging planes at the ascending and thoracic descending aorta. Blood was treated as a Newtonian and incompressible fluid with a dynamic viscosity of 4.0 mPa and a density of 1060 kg/m^3^, and the aortic walls were assumed to be rigid with no slip condition. The 3D geometries were exported into ICEM CFX (ANSYS) for mesh generation. The computational mesh consisted of 3D hexahedral cells with higher resolution in the fluid boundary layer at the wall, and mesh sensitivity test was carried out to ensure mesh independence of the simulation results.

The Navier–Stokes equations were solved numerically with a commercial finite-volume-based CFD solver (ANSYS CFX 14). The laminar-turbulent transitional version of the hybrid shear stress transport turbulence model [22], which incorporates empirical correlations to cover a range of transition mechanisms that lead to turbulent (chaotic) flow, was employed in the simulations to capture possible transitional flow in the aorta.

All simulations were carried out for three cardiac cycles to achieve a periodic solution with optimized time step and convergence criteria, and the results presented here were obtained in the third cycle. Results were analysed using post-processing software ENSIGHT 10 (CEI Inc, NC, USA). One of the most important parameters, time-averaged wall shear stress (TAWSS), was calculated by averaging the time-varying wall shear stress (WSS) over the cardiac cycle.

### Statistical analysis

Formal statistical analysis was not clinically important in such proof of concept study with small number of subjects. However for descriptive purpose, statistical analysis was performed using IBM SPSS Statistics software version 20.0 (IBM Corp., Armonk, NY, USA) and Microsoft Excel 2010. Participants’ characteristics were expressed as mean ± standard deviation (continuous variables) and as frequencies (categorical variables). Statistical significance was considered where *P* ≤ 0.05. Comparative analysis between patients and control groups was carried out using non- parametric independent samples test (Mann–Whitney U) for continuous variables, and Fisher’s exact test for categorical variables.

## Results

Four white Caucasian male patients and two age and gender-matched NC were recruited. All patients had good left ventricular (LV) function prior to surgery, and they were generally well and asymptomatic at the MRI scan date, while the NC were disease and symptoms free, independent and on no regular medication, Table 1 describes the clinical characteristics of both groups. All patients underwent elective surgery, without history of Marfan’s syndrome. Post-operatively, all patients developed atrial fibrillation which all responded to treatment and reversed back to sinus rhythm before discharge.

**Table 1:** **Demographic and clinical characteristics**

Variables	Control (n = 2)	Patients (n = 4)	***P***-value
Male [n (%)]	2 (100%)	4 (100%)	_★
Age (years)	62 ± 4	66 ± 5	0.21
Age range (years)	55- 72	59- 78	
White Caucasian [n (%)]	2 (100%)	4 (100%)	_★
DM [n (%)]	0 (0%)	0 (0%)	_★
Hypertension [n (%)]	0 (0%)	3 (75%)	0.4
Statin treatment [n (%)]	0 (0%)	3 (75%)	0.4
BMI (kg/m^2^)	30 ± 1.8	28.3 ± 2.8	0.35
Heart Rate	68 ± 13	64 ± 7	0.64
SBP (mmHg)	131 ± 1	129 ± 8	0.48
DBP (mmHg)	79 ± 1	68 ± 14	0.35
PP (mmHg)	53 ± 2	61 ± 21	0.64
MAP (mmHg)	96 ± 1	88 ± 7	0.35
Height (cm)	183 ± 5	176 ± 3	0.06
Weight (kg)	101 ± 11	90 ± 12	0.16
PVD [n (%)]	0 (0%)	0 (0%)	_★
Marfan’s syndrome	0 (0%)	0 (0%)	_★
Bicuspid aortic valve	0 (0%)	1 (25%)	_★
NYHA (pre-op.) [n (%)]			0.46
Class I	2 (100%)	2 (50%)	
Class II	0 (0%)	2 (50%)	
NYHA (post-op.) [n (%)]			_★
Class I	2 (100%)	4 (100%)	
Class II	0 (0%)	0 (0%)	
**Operative characteristics**			
Indication for surgery, dilated aortic root and			
Aortic stenosis		1 (25%)	
Aortic regurgitation		2 (50%)	
Aortic stenosis and regurgitation		1 (25%)	
Elective		4 (100%)	
CPB time (minutes)		190 ± 51	
Inotropes [n (%)]		4 (100%)	
Atrial Fibrillation [n (%)]		4 (100%)	
Blood products		4 (100%)	
> 24 intubation		2 (50%)	
Hospital stay (days)		10.5 ± 2.6	

Values are shown as n (%) for categorical variables and as mean ± SD for continuous variables. ^★^No statistics are computed. DM: diabetes mellitus; BMI: body mass index; SBP: systolic blood pressure; DBP: diastolic blood pressure; PP: pulse pressure; MAP: mean arterial blood pressure; PVD: peripheral vascular disease; NYHA: New York Heart Association; CPB: cardiopulmonary bypass.

### Morphologic parameters

The geometries of aorta from patients and NC groups are presented in Figure 1. It can be observed that the normal smooth curvature of the aorta is lost in BioValsalva^TM^ patients, this is due to the development of two angles: one at the ascending aorta level and one distal to the left subclavian artery. The cross-sectional area (*A*_*sinus*_) and maximum diameter (*D*_*sinus*_) at the aortic root level were comparable between NC and BioValsalva™ patients, 8.9 ± 1.6 vs. 7.9 ± 0.9 cm^2^, and 3.36 ± 0.3 vs. 3.18 ± 0.15 cm respectively, Table 2. This hold true at the level of proximal ascending aorta (*A*_*mean*_ and *D*_*max*_), 8.1 ± 2.2 vs. 6.5 ± 0.31 cm^2^, and 3.2 ± 0.28 vs. 2.9 ± 0.12 cm respectively.

**Table 2:** **Hemodynamic parameters**

	Control (n = 2)			Patients (n = 4)					
Cases	#1	#2	Mean	#1	#2	#3	#4	Mean	***P***-value
*A*_*mean*_ (cm^2^), ascending aorta	6.5	9.7	8.1 ± 2.2	6.6	6.4	6.3	7.0	6.5 ± 0.31	0.35
*D*_*max*_ (cm), ascending aorta	3.0	3.4	3.2 ± 0.28	2.9	2.9	2.8	3.1	2.9 ± 0.12	0.16
*A*_*sinus*_ (cm^2^), aortic sinus	7.8	10.1	8.9 ± 1.6	7.7	8.8	6.8	8.5	7.9 ± 0.9	0.35
*D*_*sinus*_ (cm), aortic sinus	3.15	3.58	3.36 ± 0.3	3.13	3.34	3.0	3.28	3.18 ± 0.15	0.35
*Q*_*max*_ (ml/s)	682	400	541 ± 199	551	650	597	473	567 ± 75	1.0
*Q*_*mean*_ (ml/s)	128	62	95 ± 46	99.8	107	98	81.4	96 ± 10	1.0
*V*_*max*_ (cm/s)	238	209	223 ± 20	240	336	340	342	314 ± 49	0.06
*A*_*orifice*_ (cm^2^)	4.23	4.58	4.40 ± 0.24	2.91	2.95	2.47	3.61	2.99 ± 0.47	0.06
*A*_*orifice*_ *\A*_*sinus*_	0.56	0.52	0.54 ± 0.03	0.48	0.39	0.38	0.42	0.42 ± 0.04	0.06
TAWSS_max_ (Pa)	19.13	15.22	17.17 ± 2.7	15.15	24	12.89	17.31	17.33 ± 4.7	0.64
Compliance (mm^2^ • mmHg^−1^ • 10^−3^)	25.6	25	25.3 ± 0.4	9.3	12.4	18.6	10.2	12.6 ± 4.2	0.06
Distensibility (mmHg^−1^• 10^−3^)	4.06	2.6	3.33 ± 1.03	1.35	1.62	2.61	1.24	1.7 ± 0.62	0.16
Stiffness index β	0.21	0.26	0.23 ± 0.03	0.52	0.35	0.32	0.52	0.43 ± 0.11	0.06

*A*_*mean*_: aortic cross-sectional area; *D*_*max*._: aortic maximum diameter; *A*_*sinus*_: sinus cross-sectional area; *D*_*sinus*_: sinus diameter; *Q*_*max*_: temporal maximum flow rate; *Q*_*mean*_: mean flow rate throughout the cardiac cycle; *V*_*max*_: spatial maximum velocity at peak flow rate; *A*_*orifice*_: effective orifice area at peak flow rate; TAWS_max_: maximum time-averaged wall shear stress.

### Hemodynamic parameters

#### Flow rate and velocity

**Figure 2:**
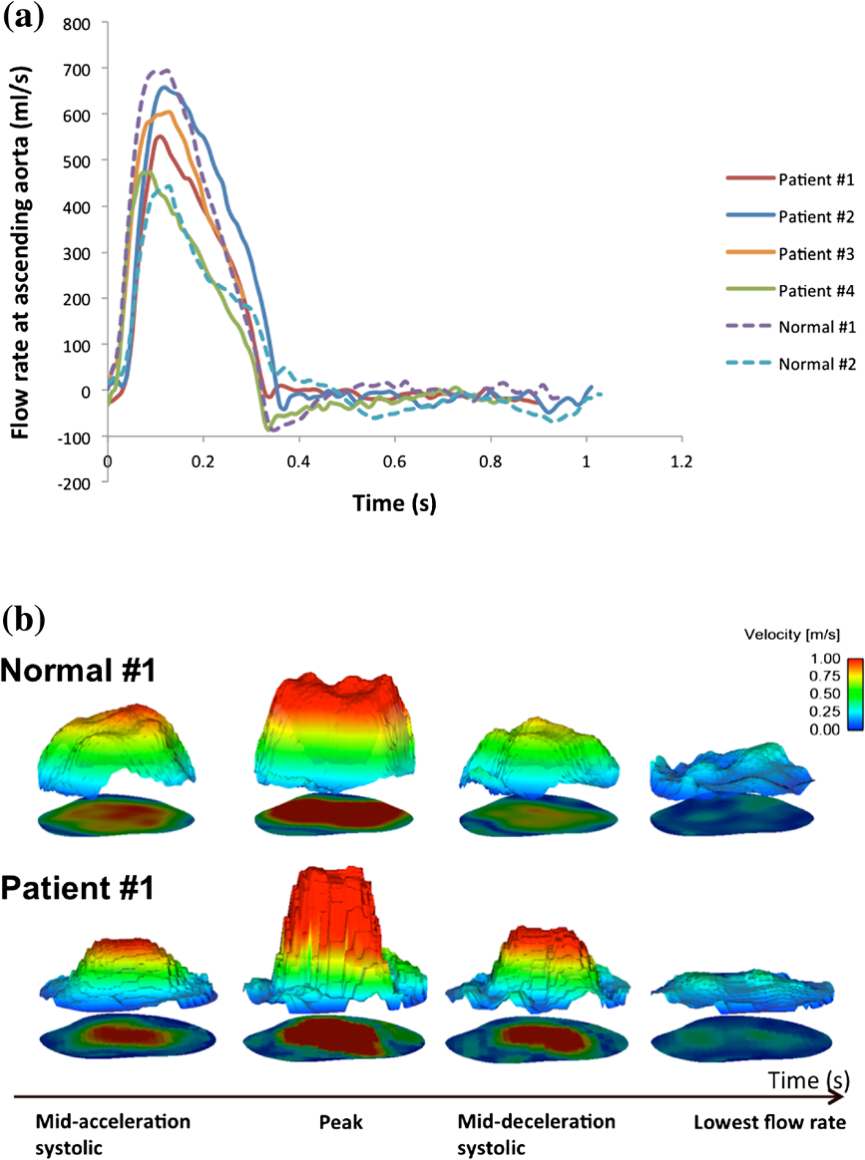
**Volumetric flow rate waveforms and spatiotemporal velocity profile.** **a)** Shows the similarity in volumetric flow rate waveforms at aortic root between the normal controls and BioValsalva™ patients. **b)** The spatiotemporal through-plane velocity profile at aortic root of subject Normal #1 (top) and Patient #1 (bottom), the velocity profile of the BioValsalva™ was steeper and with higher peak than the normal.

There was no significant difference between patients and NC in heart rate, blood pressure, height, weight and they all were functionally asymptomatic at the time of MRI scan (Table 1). Table 2 describes the main hemodynamic parameters of the NC and BioValsalva™ patients. The maximum flow rate (*Q*_*max*_) and mean flow rate (*Q*_*mean*_) throughout the cardiac cycle at the aortic root level were very comparable between NC and BioValsalva™ patients (541 ± 199 vs. 567 ± 75 ml/s) and (95 ± 46 vs. 96 ± 10 ml/s), respectively. The temporal profile of the flow rate for each case is presented in Figure 2, the shape of the flow rate waveform at the aortic root of the BioValsalva™ patients was very similar to that of NC. The maximum velocity (*V*_*max*_ (cm/s)) was higher in patients in comparison to NC (314 ± 49 vs. 223 ± 20).

#### Spatiotemporal velocity profile

**Figure 3:**
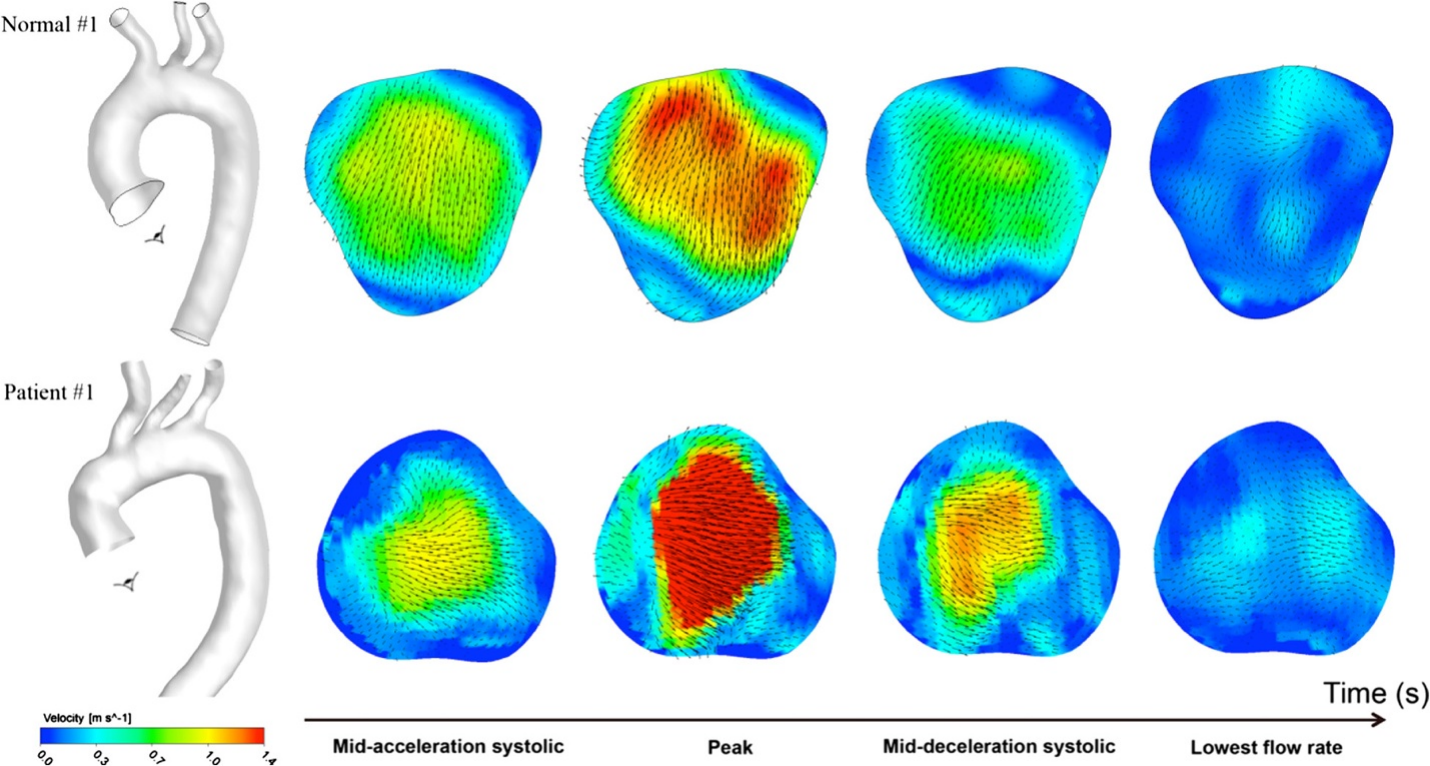
**The in-plane and the through-plane velocities at aortic root.** The in-plane velocity projected on the cross section area of aortic root (vectors) and the through-plane velocity (color contours) are visualized for subject Normal #1 (top) and Patient #1 (bottom). Although the in-plane flow is organized in the orifice area in both subjects, the BioValsalva™ patient is more likely to have swirl near the edge of orifice area.

Over the cross section area of the aortic root, the through-plane and in-plane components of flow velocity were derived from three-direction encoded flow information. The spatiotemporal velocity profile (4-time points) is shown in Figure 2. The velocity profile of the BioValsalva™ was steeper and with higher peak than the NC. The in-plane flow is usually much lower compared to the trough-plane component which is the main direction of the flow, but it could show the secondary flow patterns in the region therefore observing the presence of retrograde, vortex or separate flow. The in-plane velocity is visualized as vectors on the cross-sectional plane and the through-plane velocity colour coded for subject Normal #1 and Patient #1 (Figure 3). The in-plane flow is quite organized in the orifice area in both subjects, however the patient subject is more likely to have swirl near the edge of orifice area and relatively larger retrograde flow in the surround region.

### Effective orifice area

**Figure 4:**
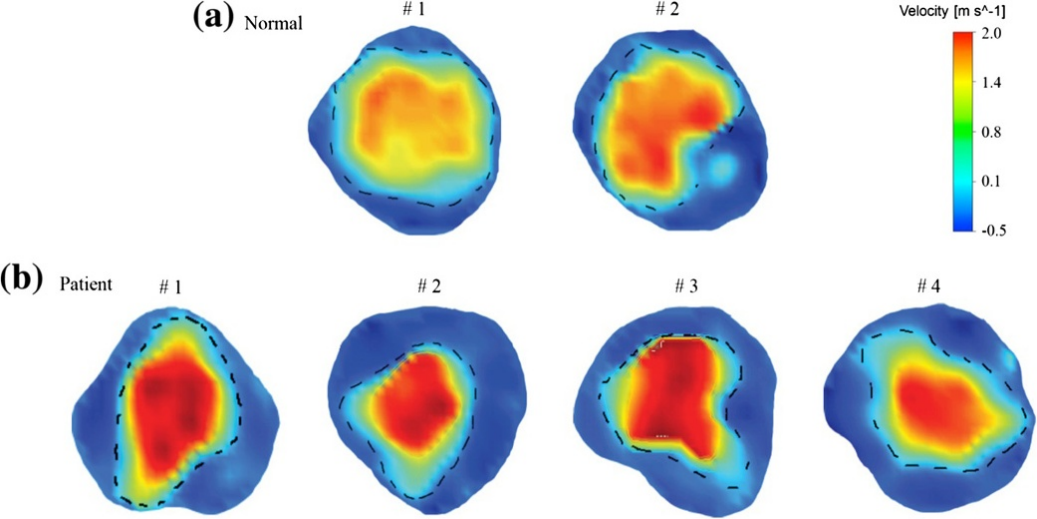
**The effective orifice area (EOA).** Colour-coded velocity map showing the EOA at peak flow rate above the valves (broken lines) for all subjects, **(a)** Normal control group and **(b)** BioValsalva™ patients group. The shapes of the EOA in BioValsalva™ are less regular and smaller.

The EOA at peak flow rate (*A*_*orifice*_ (cm^2^)) of the NC was more than 30% larger than that in BioValsalva™ patients, 4.40 ± 0.24 vs. 2.99 ± 0.47. Using the ratio of EOA to sinus cross sectional area (*A*_*orifice*_*\A*_*sinus*_) makes the difference between the two groups less evident, 0.54 ± 0.02 (control) vs. 0.42 ± 0.04 (BioValsalva™), Table 2. Visual illustration of the effective orifice area at peak flow rate above the valves was constructed (broken lines) from the colour-coded velocity map, Figure 4. As shown, the shapes in BioValsalva™ are less regular and smaller with wide margin between the orifice and edge of the sinus.

### Aortic wall elasticity

Native aortic wall is more elastic than the BioValsalva™ conduit with twofold compliance (mm^2^ • mmHg^−1^ • 10^−3^) value (25.3 ± 0.4 vs. 12.6 ± 4.2). Superior elastic properties of the native aortic wall were also confirmed by other aortic stiffness indices such as distensibility and stiffness index as shown in Table 2.

### Wall shear stress (WSS)

**Figure 5:**
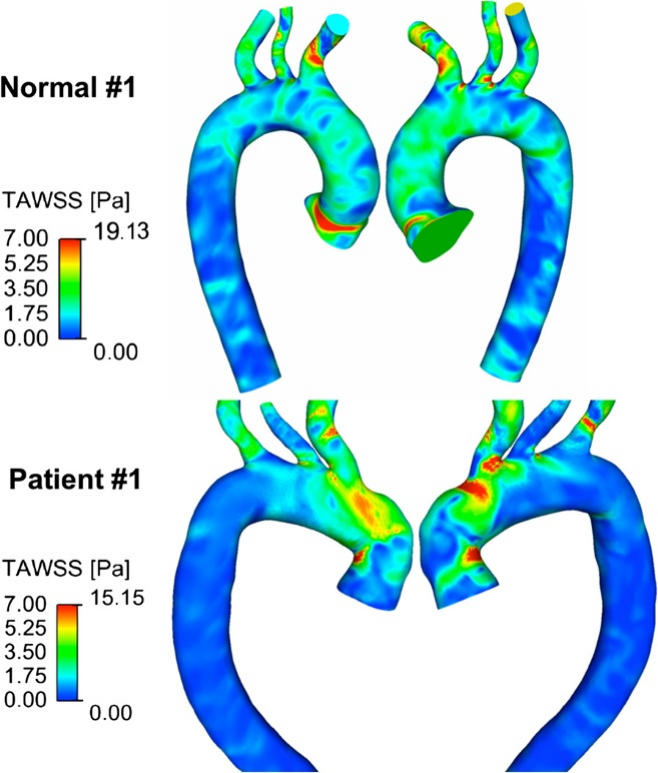
**The time-averaged Wall Shear Stress (TAWSS) for subject Normal #1 and Patient #1.** The maximum TAWSS values were comparable between the two groups, but the spatial distribution of areas with high wall shear stress was different.

The maximum time-averaged wall shear stress, TAWSS _max_ (Pa) was equivalent between the two groups, 17.17 ± 2.7 (controls) vs. 17.33 ± 4.7 (BioValsalva™ patients), Table 2. Using Normal #1 and Patient #1 as representing cases, generally, in NC, TAWSS was higher on the anterior and right segments of the aortic root and ascending aorta, while in BioValsalva™ patients it was elevated in the left segments of the ascending aorta and anterior-superior segment of the proximal aortic arch, Figure 5. The maximum TAWSS in NC was localised at the sinus area while in BioValsalva™ patients was at the outer curve of the ascending aorta just distal to the suture line, Figure 5.

### Blood flow pattern

**Figure 6:**
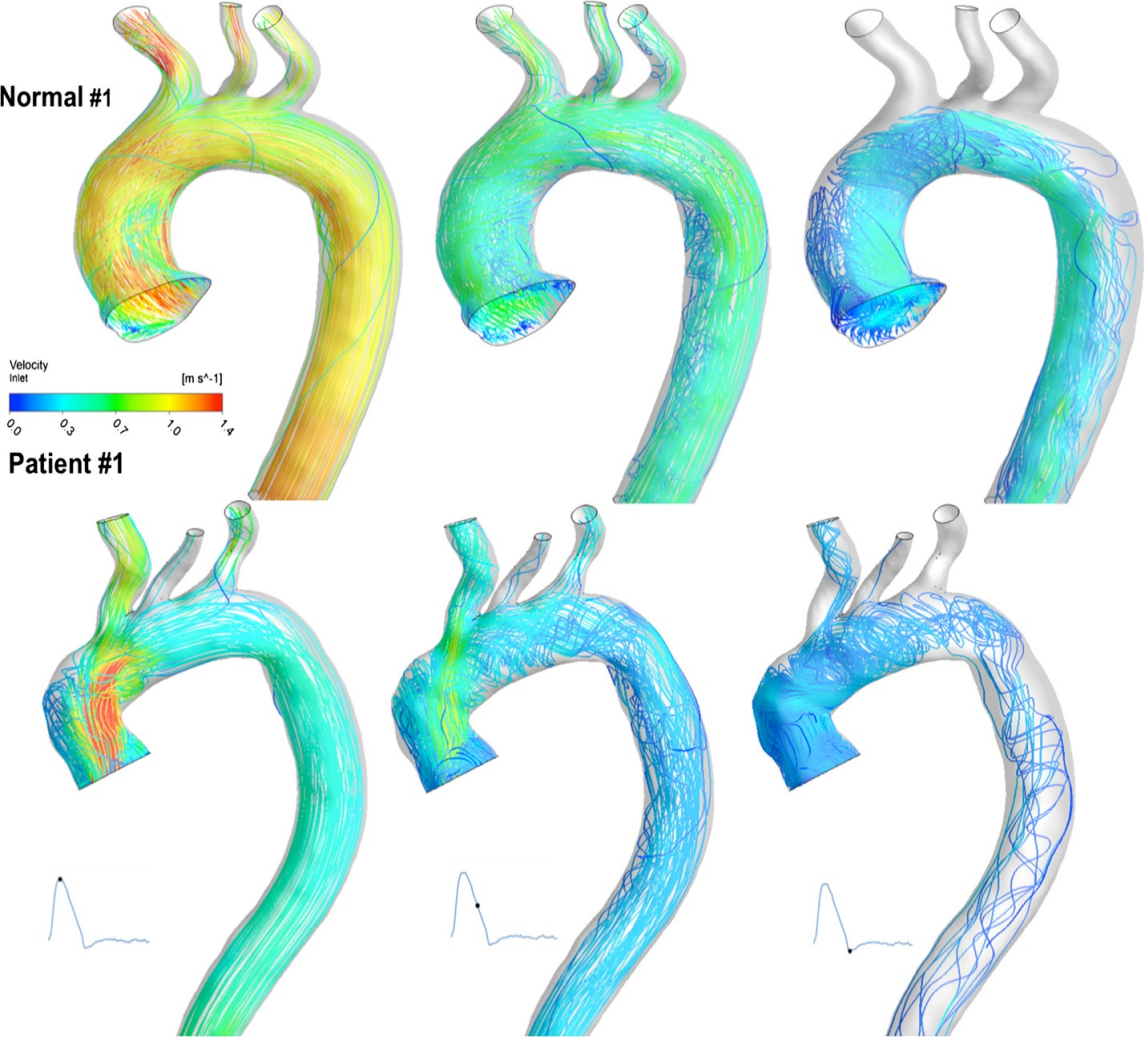
**Flow streamlines for subject Normal #1 and Patient #1.** The flow at the outer curvature of the ascending aorta was more uniform and streamlined in normal aorta in comparison to the BioValsalva™ patient.

Blood flow patterns are displayed by instantaneous flow streamlines (Figure 6). The central part of the flow was more organised and comparable between the two groups. The flow at the outer curvature of the ascending aorta was more uniform and streamlined in the NC with minimum disturbance, while in BioValsalva™ patients more disturbed flow with recirculation was noticed at the nearly right-angle that developed at the suture line.

## Discussion

Our literature search could not identify any study investigating BioValsalva™ functional performance using similar methodology. Therefore there were no previous results to compare with. The number of studies investigated BioValsalva™ is limited and focusing mainly on mortality and operative morbidity rates [13]-[16],[23]. Few of them have performed routine post-operative trans-thoracic echocardiography to assess cardiac function, trans-valvular gradient paravalvular leak, and valvular regurgitation [15],[16],[23]. Two studies assessed the EOA of earlier generation, one use CT scan [16], and the other used trans-thoracic echocardiography [13].

The design of valvular or vascular prosthesis is usually inspired by the design of the native tissue to achieve maximum possible similarity in structure and function. The availability of advanced imaging and computational technology makes the assessment of the degree of similarity between such prosthesis and normal tissue achievable. From structural design point, BioValsalva™ composite valve-conduit graft is comparable to native tissue in terms of biological valve, tricuspid design, stentless valve frame, coronary sinuses and proportionally sized conduit. From functional point, they seem very comparable to normal tissue in term of maximum flow rate (ml/s), mean flow rate, flow rate waveform (Table 2 and Figure 2). In addition, cross-sectional area and maximum diameter of aortic root and ascending aorta, were also comparable between the two groups (to some extent higher in the control group).

However, though the overall cross-sectional area and maximum diameter at the sinus level was comparable between the two groups which may indicate correct intra-operative prosthetic size selection, the EOA (*A*_*orifice*_) at peak flow rate was smaller in BioValsalva™ patients (4.40 ± 0.24 vs. 2.99 ± 0.47 cm^2^). This difference might be inevitable due the space occupied by the prosthetic valve frame and suturing mattress as indicated by the wide margin between the effective orifice and the edge of the sinus (Figure 4), it may also suggest that BioValsalva™ valve opening is not free and wide as the normal valve. Other factor that might contribute to the difference in EOA is the body size, the height (cm) of healthy control was markedly taller than the patients (183 ± 5 vs. 176 ± 3), which may also explain the differences in the aortic cross-sectional area and aortic maximum diameter (*A*_*mean*_: *D*_*max*._; *A*_*sinus*_ and *D*_*sinus*_). Two previous studies assessed the EOA of earlier generation, the results of both were smaller than what we found using MRI scan in newer generation of the prosthesis. The EOA was 2.2 ± 0.3 and 2.4 ± 0.4 cm^2^ for the 25- and 27-mm prostheses when assessed with CT scan [16], and 1.9 ± 0.3 cm^2^ when assessed with trans-thoracic echocardiography [13].

However, the global hemodynamic performance of the BioValsalva™ was not affected by this difference in the EOA. Smaller EOA has caused higher maximum velocity (cm/s) in the patients group (314 ± 49 vs. 223 ± 20), as a small orifice produces high-velocity jet into the aortic root, thus, the spatiotemporal velocity profile of the BioValsalva™ was steeper and faster than the control (Figure 2).

Another difference was in the elastic property of the vascular (aortic) conduit. As expected, the BioValsalva™ conduit is stiffer than the native aortic wall which has higher compliance and distensibility. This is because native aorta is a viable tissue and, histologically, particularly at the proximal location, is rich in elastin tissue [24]. Whether or not the rest of the normal aorta can counterbalance this stiffness to deliver steady normal flow at the periphery need to be assessed in future study.

Flow streamlines demonstrated that the flow pattern was also comparable between the two groups apart from the more disturbed and helical flow that occurs at the outer curvature of the nearly right-angle turn at the ascending aorta level. From the site and level (high above the valve) of this helical flow, it seems to be related to the sharp angle in the flow pathway, makes the surgical technique more accountable to this difference than the prosthetic design. Though the TAWSS _max_ (Pa), were also comparable between the two groups, the spatial distribution of areas with high wall shear stress was different between the two groups. By examining the close correlation between flow streamlines and TAWSS maps (Figures 5 and 6), the difference in the TAWSS could be due to: the difference in the maximum flow velocity (high-speed jet in the BioValsalva™) through the valve would result in higher shear stress on the aortic wall; the development of nearly right-angle turn at the ascending aorta near the anastomosis (suture) line which might cause more disturbance to the aortic blood flow.

Our findings are in line with previously published results using similar technology in normal controls and xenograft aortic root replacement patients (Medtronic Freestyle, Medtronic Inc, Minneapolis, MN, USA), which validates our results and methodology [18]. The shapes of the flow rate waveforms were also very similar between the studies. Interestingly, the mean EOA was also comparable between the two studies for both patients and healthy controls, and the spatiotemporal velocity map of our control and BioValsalva™ were also similar to the published normal and xenograft respectively [18].

### Limitations

One could argue the small number of participants recruited, however, considering the proof of concept nature of the study and the significant time and effort needed for imaging data reconstruction and computational modelling, bigger number of subjects would not be necessary. Though unlikely to change the general direction of the results, coincidently, all BioValsalva™ patients were male, therefore the effect of gender on the results cannot be confirmed. Another limitation is that coronary flow rate was not assessed, this could shed some light on whether the difference in aorta geometry and conduit compliance can affect coronary circulation.

## Conclusions

Combined Cardiac MRI and Computational Modelling Technology is an effective method of assessing valvular and vascular prosthesis from structural and functional point of view and it should be considered as a part of validation assessment of any new prosthesis. BioValsalva™ composite valve-conduit prosthesis is potentially comparable to native aortic valve and aorta in structural design and in many functional hemodynamic patterns, further larger study to confirm these finding is desired. The functional differences between BioValsalva™ and normal aortic valve are mainly in the elasticity of the vascular conduit, therefore future design development should focus on this aspect. Surgeons should pay more attention to the surgical technique they use in sizing and suturing to maximise the reestablishment of normal smooth aortic curvature to prevent unfavourable flow characteristics. Future larger studies using such technology should also consider exploring the clinical impact of unfavourable hemodynamic parameters and the effect of proximal hemodynamic on distal circulation.

## Authors’ information

Emaddin Kidher and Zhuo Cheng Sharing first authorship.

**Institution of work**: Imperial College London and Imperial College Healthcare NHS Trust, London, UK.

## Authors’ original submitted files for images

Below are the links to the authors’ original submitted files for images. Authors’ original file for figure 1 Authors’ original file for figure 2 Authors’ original file for figure 3 Authors’ original file for figure 4 Authors’ original file for figure 5 Authors’ original file for figure 6
