# Supplementary figures and images for: In-vivo assessment of the morphology and hemodynamic functions of the BioValsalva™ composite valve-conduit graft using cardiac magnetic resonance imaging and computational modelling technology
Source: J Cardiothorac Surg. 2014 Dec 9;9:193. doi: 10.1186/s13019-014-0193-6 (PMC4263057; doi:10.1186/s13019-014-0193-6)

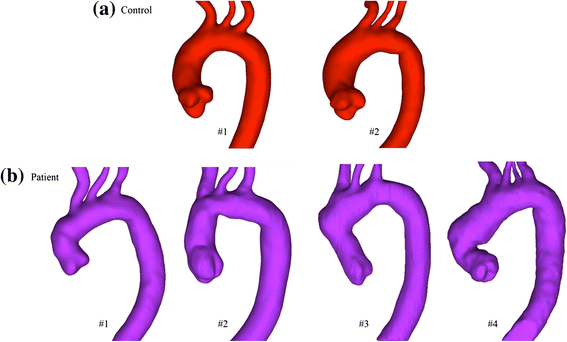

Supplement: Supplementary file 1 — Authors’ original file for figure 1 [file 13019_2014_193_MOESM1_ESM.gif]

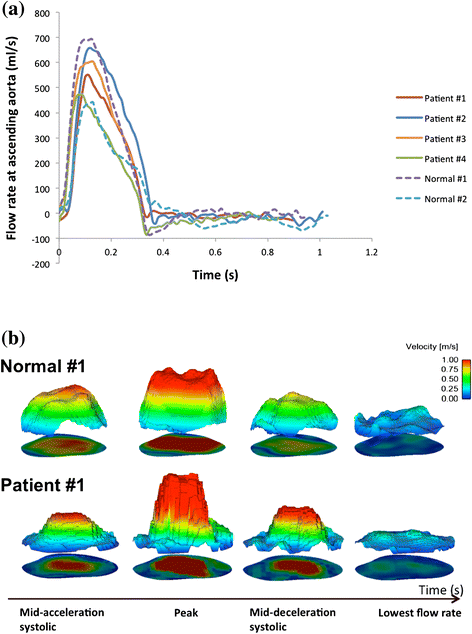

Supplement: Supplementary file 2 — Authors’ original file for figure 2 [file 13019_2014_193_MOESM2_ESM.gif]

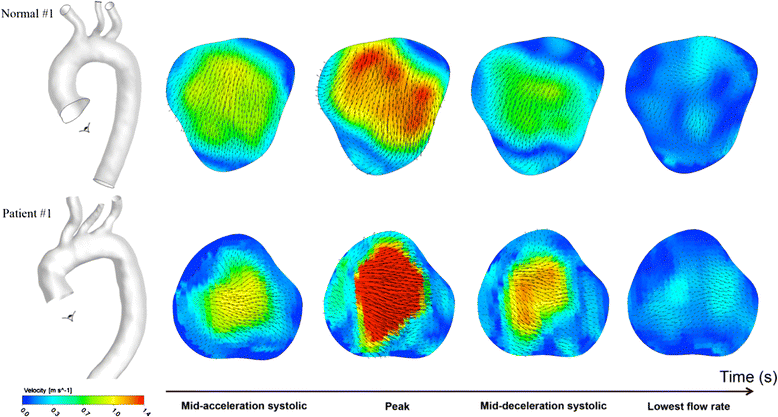

Supplement: Supplementary file 3 — Authors’ original file for figure 3 [file 13019_2014_193_MOESM3_ESM.gif]

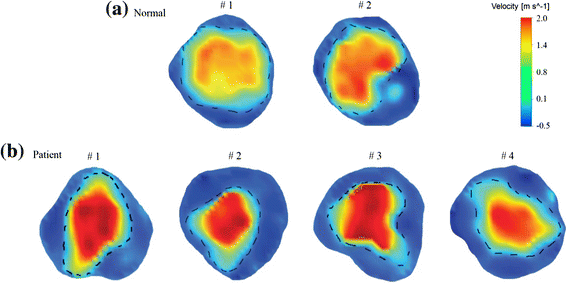

Supplement: Supplementary file 4 — Authors’ original file for figure 4 [file 13019_2014_193_MOESM4_ESM.gif]

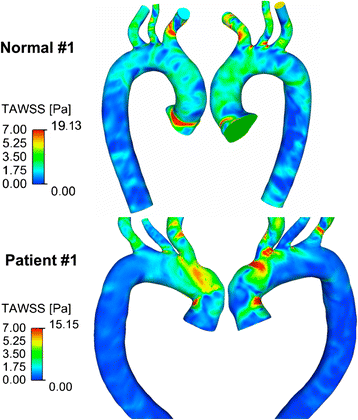

Supplement: Supplementary file 5 — Authors’ original file for figure 5 [file 13019_2014_193_MOESM5_ESM.gif]

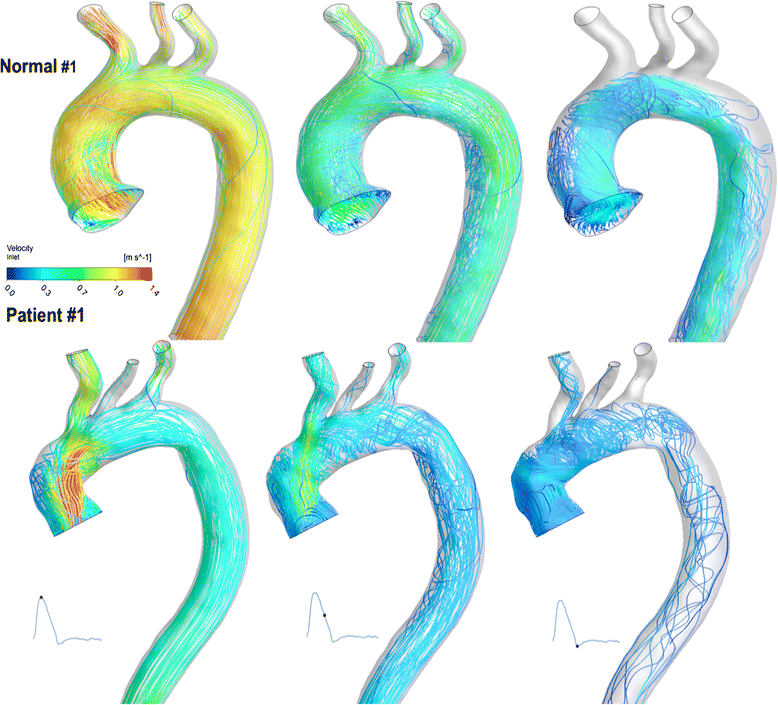

Supplement: Supplementary file 6 — Authors’ original file for figure 6 [file 13019_2014_193_MOESM6_ESM.gif]
